# A Randomised Clinical Trial to Compare the Efficacy and Safety of Dexmedetomidine-Ropivacaine Versus Fentanyl-Ropivacaine for Epidural Labour Analgesia

**DOI:** 10.7759/cureus.68076

**Published:** 2024-08-29

**Authors:** Reshma R S, Shivanand L Karigar, Shreedevi Kori, Pratibha S D

**Affiliations:** 1 Anaesthesiology, Shri B. M. Patil Medical College Hospital and Research Center, Bijapur Lingayat District Educational (Deemed to be University), Vijayapura, IND; 2 Obstetrics and Gynaecology, Shri B. M. Patil Medical College Hospital and Research Center, Bijapur Lingayat District Educational (Deemed to be University), Vijayapura, IND

**Keywords:** labour analgesia, epidural, ropivacaine, adjuvant, dexmedetomidine, fentanyl

## Abstract

Background

An epidural block is a superior potent approach to labour analgesia, and ropivacaine combined with fentanyl has been successfully practised for it. Dexmedetomidine, as a novel form of labour analgesia, must be researched further. Our study results give insight into the epidural block and come up with a pioneering approach for labour analgesia.

Methodology

A total of 68 parturients were assigned to two equal groups and received either dexmedetomidine with ropivacaine (Group RD) or fentanyl with ropivacaine (Group RF). Parturients received a loading dose and maintenance was given using a patient-controlled analgesia (PCA) pump. Analgesia onset time, labour duration, rescue dose requirement, and Visual Analogue Scale (VAS) pain scores were noted. Appearance, Pulse, Grimace, Activity and Respiration (APGAR) scores of newborns, Ramsay Sedation Scale (RSS) scores of mothers, and maternal side effects were observed.

Results

Group RD showed a shorter onset time of analgesia (group RD: 12.50 ± 1.31 minutes vs. group RF: 15.26 ± 1.46 min), less local anaesthetics requirement (group RD: 47.54 ±5.37 ml vs. group RF: 59.05 ± 6.62 ml), less number of bolus doses (group RD: 0.15 ± 0.36 vs. group RF: 1.21 ± 0.95), and shorter duration from the epidural administration to the delivery (group RD: 312.97 ± 42.40 minutes vs. group RF: 345.94 ±14.67 minutes) than group RF. VAS values of the RD group were significantly less than the RF group. The RSS scores were comparably low in both groups, and excessive sedation was not seen in any group. Newborn APGAR values were comparably on the higher side in the two groups. Adverse effects were observed in the two groups, like hypotension, nausea/vomiting, bradycardia, shivering, and pruritus, which were insignificant.

Conclusion

The RD group showed an improved analgesic effect with a quicker onset of action, reduced requirement of local anaesthetics, and lower VAS scores compared to the RF group. With ropivacaine, dexmedetomidine shows more efficacy than fentanyl during epidural block and is a safe alternative for labour pain management.

## Introduction

Donald D. Moir (the father of labour analgesia) wrote, "The delivery of the infant into the arms of a conscious and pain-free mother is one of the most exciting and rewarding moments in medicine" [1].

Some women breeze through giving birth, while most women must go through the most painful moments during childbirth. Delivery pain can cause a rise in maternal stress hormones and blood pressure, hyperventilation, reduced foetal oxygen transport, and mental distress to the mother. Most women find delivery pain to be among their most agonising experiences, and it may be harmful to the mother and the baby [2]. Pain influences the progress of labour and the foetal outcome. Therefore, reducing pain during labour will help to enhance perinatal and mother outcomes. Healthcare professionals are constantly searching for methods to improve analgesic efficacy and decrease the harmful effects of labour pain.

The most efficacious method of labour analgesia is an epidural blockade, which is favoured due to its benefits, such as customisable pain relief, minimal motor block, and good safety profile [3]. Due to the use of anaesthetics, epidural labour analgesia may have unfavourable effects, including maternal hypotension, prolonged second stage of labour, and urine retention [4]. For this reason, choosing the right anaesthetic is crucial for epidural labour analgesia. Ropivacaine is a long-acting amide local anaesthetic typically utilised in clinical practice for delivery with fewer side effects [5]. Synthetic opioids like lipid-soluble sufentanil and fentanyl can bring up the potency of local amide anaesthetics by altering the minimum potency [6].

Studies have shown that local anaesthetics ropivacaine and fentanyl together work well to relieve pain during epidural labour analgesia. The amount of pain and the need for rescue analgesics are both lowered. Side effects seen with opioids might include reduced foetal heart rate variability, nausea, vomiting, pruritus, respiratory depression, and urine retention [7]. Dexmedetomidine has been successful in the management of epidural labour analgesia with fewer adverse effects. As an α2-adrenoceptor agonist and non-opioid drug, dexmedetomidine has strong pain-relieving and anxiety-relieving properties without slowing down breathing [8]. More research needs to be done to find out the pros and cons of using dexmedetomidine along with ropivacaine in a new way to treat labour pain with an epidural block.

Our trial was uniquely designed to determine the safety and efficacy of using ropivacaine along with dexmedetomidine compared to fentanyl for epidural labour analgesia. The findings of our study not only shed light on the epidural block but also present a pioneering approach to pain management during childbirth. 

Aims and objectives

Our research aimed to compare the efficacy and safety of dexmedetomidine-ropivacaine versus fentanyl-ropivacaine for epidural labour analgesia. The study objectives were to find out how dexmedetomidine and fentanyl compare as epidural adjuvants in terms of the time of onset of analgesia, duration from epidural administration to delivery of baby, how much rescue analgesic doses were needed, the Visual Analogue Scale (VAS) values, the newborn Appearance, Pulse, Grimace, Activity and Respiration (APGAR) scores, the Ramsay Sedation Scale (RSS) values, and maternal adverse effects observed.

## Materials and methods

Study subjects

We conducted a randomised clinical trial in Shri B. M. Patil Medical College Hospital and Research Centre, Bijapur Lingayat District Educational (BLDE) (Deemed to be University, DU), Vijayapura, Karnataka, within the Labour Room Complex, over a year after receiving approval from the Institutional Ethical Committee, BLDE (DU) (approval letter: IEC/784/2022-23). Our study was registered under the Clinical Trials Registry of India under the registration number CTRI/2023/06/053707. Primigravidas with cephalic presentation, American Society of Anaesthesiologists (ASA) grades I and II, single foetus, mothers between the ages of 19 and 35 years, gestation age ≥ 35 weeks, and a cervical dilatation of ≥ 4 cm were the inclusion criteria. Hypotension/hypertension, endocrine disorders, foetal compromise, preterm gestation, coagulopathies, study agent allergies, and contraindications to epidural analgesia were the exclusion criteria.

Sample size

The sample size calculation was performed using G*Power version 3.1.9.4 software (Heinrich Heine University Düsseldorf, Düsseldorf, Germany) based on the mean gestational age for Group RS (ropivacaine + sufentanil) (37.41 weeks, SD = 3.94) and Group RD (ropivacaine + dexmedetomidine) (39.10 weeks, SD = 0.91) as reported by Li et al. [9]. For this investigation to attain a power of 80% with a 5% level of significance (two groups) to identify a difference in means, an overall sample size of 68 (for two groups of 34 with matching sample sizes) was needed.

Group allocation

A separate investigator employed a computer-generated randomisation table to designate a total of 68 parturient women to two equal groups. The envelopes containing the groups had been sealed and released briefly prior to the procedure. The 34 allotted candidates in Group RD were administered 0.1% ropivacaine with 0.5 mcg/ml dexmedetomidine, while 34 allotted women in Group RF were given 0.1% ropivacaine with 2 mcg/ml fentanyl [9].

Procedure

Routine investigations were conducted, informed consent was obtained in writing, and a pre-anaesthetic examination was performed. In order to ensure that any possible consequences of the anaesthetic approach were eliminated, all procedures were performed by the same anaesthesiologist. The baseline vitals, including non-invasive blood pressure, heart rate, and room air saturation, were recorded and continuously monitored. Venous access was secured. When cervical dilatation reached 4 cm or more, the parturient was positioned in a sitting posture, and an 18 G Tuohy needle was used to introduce a catheter into the epidural space at the level of L2/L3 intervertebral spaces. After 3 ml of 2% lignocaine with adrenaline was given as a test dose through the epidural catheter, parturients were administered 10 ml of either 0.5 mcg/ml dexmedetomidine (Group RD) or 2 mcg/ml fentanyl (Group RF) combined with 0.1% ropivacaine as a loading dose. A maintenance dose of analgesia was administered using a Mindray Patient Controlled Analgesia (PCA) pump (Mindray Medical International Limited, Shenzhen, Guangdong, China). The PCA pump was permitted to administer a continuous infusion of 7 ml/hour of the same mixed drug solution. A rescue dosage of 7 ml was set as well for patients who exhibited a VAS assessment score of five or higher and were experiencing inadequate pain relief, with a predetermined lockout period of 25 minutes. The maximal flow rate was determined to be 25 ml/hour.

Data collection

Our study assessed VAS values before epidural insertion as the baseline VAS and at five, 10, 20, 30, 60, 90, and 120 minutes after the initial bolus drug combination was administered. The time of the loading dose was taken as zero minutes. The interval between the time of the loading dose administration and the mother displaying a VAS score less than three was considered the onset of analgesia. The time from the end of epidural administration to the delivery of the baby was considered the duration of labour. Rescue analgesia bolus doses in each group were monitored.

The side effects were respiratory depression: oxygen saturation falls below 90% on room air, hypotension: reduction in blood pressure by more than 20% from the initial value or blood pressure below 90/60 mmHg, and bradycardia: decrease in heart rate by more than 20% from the initial value or below 60 beats per minute. The patients were under observation, and all adverse symptoms were recorded and managed. Excessive sedation was defined as an RSS value of more than 4 and recorded every hour. Foetal monitoring was done continuously using a non-stress test. Following childbirth, the continuous infusion was discontinued, and the epidural catheter was taken out. The neonate status was evaluated using the APGAR score.

Statistical analysis

A statistical tool for the social sciences, IBM SPSS Statistics for Windows, Version 20, (Released 2011; IBM Corp., Armonk, New York, United States), was used to conduct the statistical analysis after the collected data was put into a Microsoft Excel sheet (Microsoft Corporation, Redmond, Washington, United States). The mean, standard deviation, count, percentages, and graphs are displayed along with the results. The Mann-Whitney U test was employed for variables that were not normally distributed, and the chi-square test was used to examine categorical variables between the two groups. If the p-value was less than 0.05, it was considered statistically significant.

## Results

**Figure 1:**
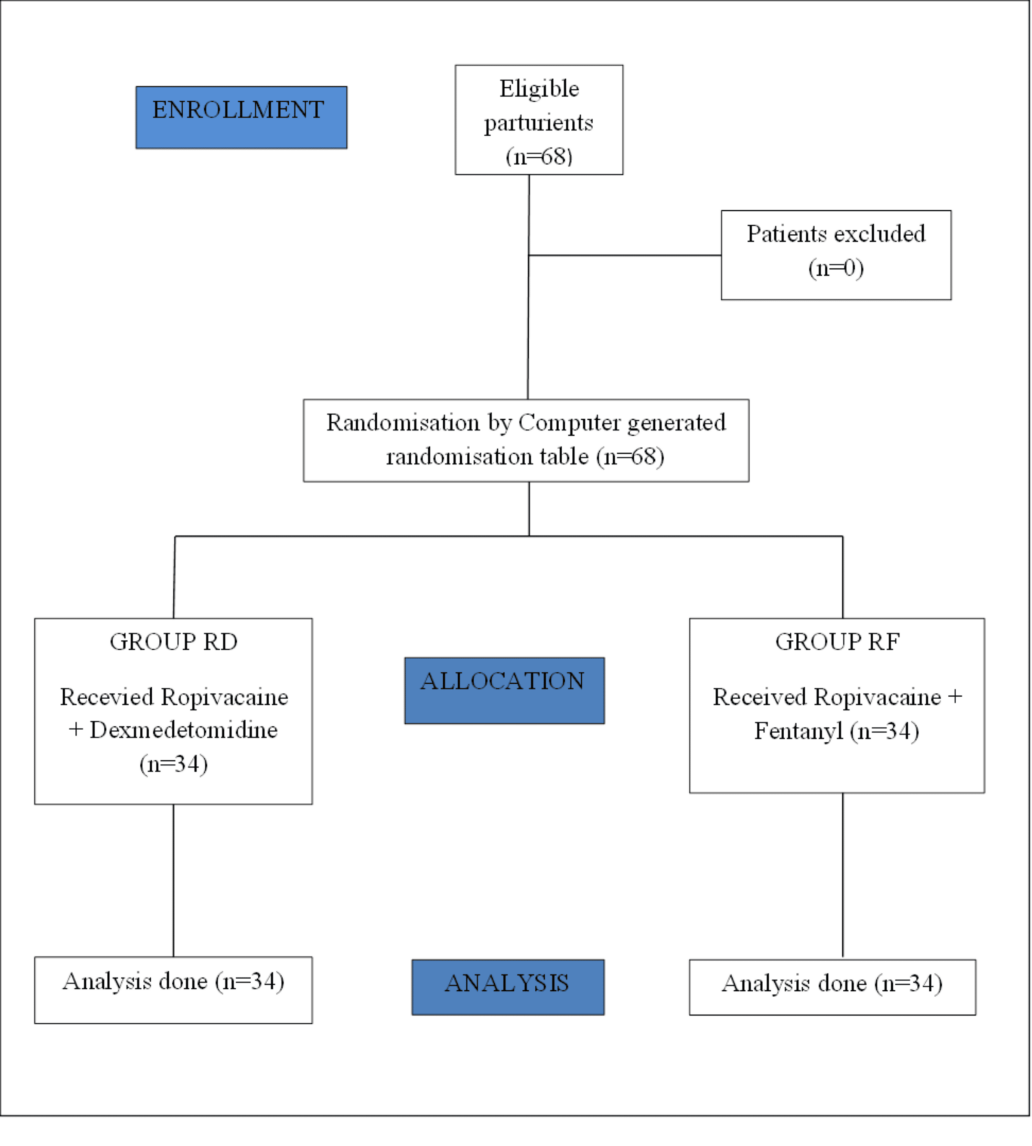
CONSORT flow chart n: number of patients; RD: ropivacaine with dexmedetomidine; RF: ropivacaine with fentanyl; CONSORT: Consolidated Standards of Reporting Trials

This randomised clinical trial consists of 68 patients divided into two groups of 34 with ropivacaine with dexmedetomidine (group RD) and ropivacaine with fentanyl (group RF) by a computer-generated randomisation table for further analysis (Figure 1).

Table 1 describes the demographic variables among group RD and group RF. Demographic variables in both groups, including period of gestation, height, weight, and body mass index, were comparable (p>0.05).

**Table 1:** Demographic parameters in the two groups RD: ropivacaine with dexmedetomidine; RF: ropivacaine with fentanyl; SD: standard deviation

Demographic data	Group RD		Group RF		Mann-Whitney U test	p-value
	Mean	SD	Mean	SD		
Period of gestation (weeks)	39.12	1.29	38.79	1.25	516.50	0.43
Height (cm)	152.44	3.39	151.24	3.47	457.00	0.14
Weight (kg)	64.44	6.51	61.59	5.96	451.50	0.12
BMI (kg/m2)	27.62	2.33	26.85	2.19	498.00	0.33

Table 2 presents a comparison of VAS scores between Group RD and Group RF. The difference in VAS values was statistically significant at five, 10, 30, 60, 90, and 120 minutes (p<0.05). The two groups were comparable in VAS scores at baseline and 20 minutes, indicating statistical insignificance at these time points (p>0.05).

**Table 2:** Comparison of visual analogue scale (VAS) values *statistically significant (p<0.05) RD: ropivacaine with dexmedetomidine; RF: ropivacaine with fentanyl; SD: standard deviation

Time point	Group RD		Group RF		Mann-Whitney U test	p-value
	Mean	SD	Mean	SD		
Baseline	9.03	5.87	8.06	0.74	527.00	0.937
At five minutes	5.76	0.78	6.21	0.64	397.50	0.016*
At 10 minutes	3.94	0.69	5.12	0.73	168.00	0.001*
At 20 minutes	2.59	0.50	2.71	0.46	510.00	0.314
At 30 minutes	2.59	0.50	3.09	0.45	324.00	0.001*
At 60 minutes	2.47	1.02	3.47	0.56	191.00	0.001*
At 90 minutes	2.74	0.86	3.68	0.68	240.00	0.001*
At 120 Min	3.41	0.82	3.88	0.84	410.00	0.027*

Table 3 shows the comparison of analgesia onset time, local anaesthetic requirement, bolus frequency, and duration of labour in RD and RF groups. In group RD, the onset time of analgesia was 12.50 ± 1.31 minutes, while in group RF, it was 15.26 ± 1.46 minutes. In group RD, the total volume of local anaesthetic solution required was 47.54 ± 5.37 ml, while in group RF, it was 59.05 ± 6.62 ml. In group RD, the bolus frequency was 0.15 ± 0.36, while in group RF, it was 1.21 ±0.95. In group RD, the duration between the epidural administration and delivery of the baby was 312.97 ± 42.40 minutes, and in group RF, it was 345.94 ± 14.67 minutes. Group RD showed a shorter time of onset of analgesia, lesser requirement for local anaesthetics, lesser bolus frequency and shorter duration from epidural administration to delivery than group RF, indicating statistical significance (p<0.05).

**Table 3:** Comparison of analgesia onset time, total volume of drug, bolus frequency, and duration of labour *statistically significant (p<0.05) RD: ropivacaine with dexmedetomidine; RF: ropivacaine with fentanyl; SD: standard deviation

Variable	Group RD		Group RF		Mann-Whitney U test	p-value
	Mean	SD	Mean	SD		
Onset Time of analgesia (minutes)	12.50	1.31	15.26	1.46	101.00	0.001*
Total volume of drug (ml)	47.54	5.37	59.05	6.62	103.00	0.001*
Bolus frequency	0.15	0.36	1.21	0.95	217.50	0.001*
Duration of labour (minutes)	312.97	42.40	345.94	14.67	225.50	0.001*

Table 4 depicts the correlation of APGAR scores of newborns in the RD group and the RF group. At one minute, an APGAR score of 7.74 was found in the RD group and 7.79 in the RF group. At five minutes, a score of 8.82 was noted in the RD group and 8.85 in the RF group. The two groups had comparable APGAR values at both time points, indicating statistical insignificance (p>0.05).

**Table 4:** Comparison of APGAR scores of newborns RD: ropivacaine with dexmedetomidine; RF: ropivacaine with fentanyl; SD: standard deviation; APGAR: Appearance, Pulse, Grimace, Activity and Respiration

Time point	Group RD		Group RF		Mann-Whitney U test	p-value
	Mean	SD	Mean	SD		
At one minute	7.74	0.51	7.79	0.48	545.00	0.565
At five minutes	8.82	0.39	8.85	0.36	561.00	0.744

Table 5 depicts the comparison of side effects among mothers in groups RD and RF. In our study, pruritus was not detected in any woman in group RD (0.0%), but it was detected in two women in group RF (5.9%). Hypotension was seen in five women in group RD (14.7%) and two women in group RF (5.9%). Nausea or vomiting was seen in three women in group RD (8.8%) and five women in group RF (14.7%). Bradycardia was seen in three women in group RD (8.8%) and none in group RF (0%). Shivering was found in three women in group RD (8.8%) and three women in RF (8.8%). Group RD showed hypotension and maternal bradycardia more than group RF, which was statistically insignificant (p>0.05). Group RF showed pruritus and nausea/vomiting more than group RD, which was statistically insignificant (p>0.05). Both groups are comparable in shivering (p>0.05). Respiratory depression and excessive sedation were not observed in any patient in either group RD or group RF throughout labour. RSS scores were observed to be less than 4 in both groups at all time periods after epidural administration.

**Table 5:** Comparison of maternal side effects RD: ropivacaine with dexmedetomidine; RF: ropivacaine with fentanyl

Maternal adverse event	Group RD	Group RF	Chi-Square test	p-value
Pruritus	0 (0.0%)	2 (5.9%)	2.06	0.151
Hypotension	5 (14.7%)	2 (5.9%)	1.43	0.231
Nausea/vomiting	3 (8.8%)	5 (14.7%)	0.56	0.452
Sedation and respiratory depression	0 (0.0%)	0 (0.0%))	0	1.000
Bradycardia	3 (8.8%)	0 (0.0%)	3.138	0.076
Shivering	3 (8.8%)	3 (8.8%)	0.00	1.000

## Discussion

Labour analgesia is a challenging path with satisfying outcomes. Labour analgesia has evolved from chloroform in the 19th century to automated central neuraxial delivery systems in the 21st century [10]. The ideal drug for pain management during labour should provide a desirable sensory blockage with no motor block, no tachyphylaxis, and unintended overdose or accidental intravenous delivery should have a suitable safety range [11]. Epidural analgesia is favoured for labour pain management due to its benefits such as customisable pain relief, minimal motor block, and a high safety profile, making it a preferred option for women in labour. Ropivacaine is a local anaesthetic that is typically utilised in clinical practice for delivery. In order to prevent motor block, augment the analgesic action, lower the dosage of local anaesthetics, and lessen the likelihood of associated adverse effects, it is currently standard practice to combine local anaesthetics with adjuvant agents [11]. To provide epidural labour analgesia, fentanyl has frequently been utilised as an adjuvant to ropivacaine. Fentanyl, as an opioid, is known to cause side effects, including headaches, vomiting, urine retention, pruritus, and respiratory depression [7]. Dexmedetomidine, an α2-adrenoceptor agonist, has been effectively utilised recently for epidural labour analgesia [8]. Dexmedetomidine exhibits highly selective, sedative, anxiolytic, sympatholytic, and analgesic effects, yet it might result in bradycardia and hypotension [7,12]. In our study, we investigated the safety and efficacity of using ropivacaine along with dexmedetomidine compared to using ropivacaine along with fentanyl for labour pain management.

In a study conducted by Cheng et al. on the analgesic effects of dexmedetomidine and sufentanil combined with ropivacaine in epidural analgesia during labour, it was found that the dexmedetomidine group showed lower values than the sufentanil group in the VAS (p<0.05) [13]. These results were similar to our study, where the dexmedetomidine group exhibited lesser VAS values compared with the fentanyl group at most of the time points after epidural placement, which was statistically significant. VAS values are important parameters for assessing labour pain [14]. The results indicate that the usage of Dexmedetomidine along with a local anaesthetic showed a higher analgesic effect compared to fentanyl as an adjuvant. Analgesia is the outcome of blocking the pain impulse going through the epidural space nerve, which mostly starts within minutes after epidural administration [4]. Koraki et al. derived that the onset time of epidural dexmedetomidine with ropivacaine was ~15 minutes [15]. Bang et al. suggested that the onset time of epidural fentanyl with ropivacaine varied from eight minutes to 19 minutes, according to increasing doses of fentanyl [16]. Our study noticed a shorter onset time of 12.50 ± 1.31 minutes in the dexmedetomidine group. In contrast, the fentanyl group showed an onset of analgesia in 15.26 ± 1.46 minutes from the time of epidural administration.

Dexmedetomidine acts as an analgesic by activating the spinal cord α2 receptors, causing an extended analgesic impact [17,18]. In the Li et al. study, the parturient women in group ropivacaine with dexmedetomidine required a lesser volume of injection and lesser local anaesthetic doses compared with group ropivacaine and sufentanil (p<0.05) [9]. Similarly, in our study, the volume of drug requirement was significantly reduced in the RD group compared to the RF group. A few studies suggest that dexmedetomidine, an α2-adrenoceptor agonist, might shorten the first stage of labour by causing contractions in the smooth muscles in the uterus [19]. A study done by Zhang et al. compared the effects of dexmedetomidine (group D) and sufentanil (group S) and put forward that the first-stage labour duration was shorter in group D (p<0.05) [19]. Our study also observed that the duration from epidural administration to delivery of the baby was shorter in group RD than in group RF, which was statistically significant.

Dexmedetomidine is known to produce sedation by acting on the locus coeruleus [20]; therefore, monitoring sedation levels became important for the study. RSS scores are good for evaluating and monitoring the effects of sedative drugs [21]. Li et al. suggested that there is no significant difference in RSS scores in the dexmedetomidine and fentanyl groups [22]. In our study, similar results were found. No significant variation in RSS values was observed across the two groups, and no evidence of profound sedation was recorded. When used appropriately, the APGAR score serves as a tool for standardised assessment and defines the newborn status soon after birth. Additionally, it offers a way to record the shift from foetal to neonatal state and describes the response to resuscitation [23]. In the study by Fan et al., APGAR scores of newborns were high in both the dexmedetomidine and sufentanil groups [24]. Similarly, in our study, newborn APGAR scores at one and five minutes remained high in the RD and RF groups.

It is postulated that opioids cause several adverse effects, like pruritus, by the presence of an itch centre in the central nervous system, medullary dorsal horn activation, antagonism of inhibitory transmitters, modulation of the serotonergic pathway, and according to a theory, the linking of pruritus with pain [25,26], even though the exact reason is unclear. Gao et al. stated that, compared with opioids, using dexmedetomidine as a local anaesthetic decreased the incidence of pruritus, nausea, and vomiting without increasing the incidence of adverse events [27]. On the contrary, our study showed side effects in both groups even though no statistical significance was derived. Maternal hypotension (14.7%), nausea/vomiting (8.8%), bradycardia (8.8%), and shivering (8.8%) were observed in group RD, while pruritus (5.9%), hypotension (5.9%), nausea/vomiting (14.7%), and shivering (8.8%) were seen in group RF.

Our study has limitations in specific ways. First, other dosages should be tested in future trials as our research evaluated the safety and efficacy of 0.1% Ropivacaine in combination with 0.5 mcg/ml dexmedetomidine and 2 mcg/ml fentanyl. Second, we conducted a single-centre research trial. Extensive multi-centre research should be conducted to confirm findings. Third, only primigravidas were investigated in our study. Further studies with multigravida on larger groups can be done to test our findings.

## Conclusions

Dexmedetomidine and fentanyl along with ropivacaine produced excellent analgesia and satisfaction for the parturient women. However, the RD group displayed a faster onset of action, reduced local anaesthetic demand and lower VAS scores than the RF group, which indicated better analgesic efficacy. The two groups did not show any evidence of profound sedation or low newborn APGAR scores. The incidence of adverse effects in the parturient women was low in both RD and RF groups. Thus compared to fentanyl, dexmedetomidine as an adjuvant displayed better efficacy in labour pain management. Dexmedetomidine along with ropivacaine can be safely used for labour analgesia. 
